# Intractable Neuromuscular Blockade Resistant to Sugammadex Rescue Administration: A Case Report

**DOI:** 10.7759/cureus.91565

**Published:** 2025-09-03

**Authors:** Vinh T Nguyen, Jason Cosman, Govind Rajan

**Affiliations:** 1 School of Medicine, California University of Science and Medicine, Colton, USA; 2 Anesthesiology and Perioperative Medicine, University of California, Irvine (UCI) Health, Orange, USA

**Keywords:** paralysis (chemically induced), recurarization, residual neuromuscular blockade, rocuronium, sugammadex

## Abstract

We describe the failure of sugammadex when used as a rescue agent, after neuromuscular blockade with rocuronium was insufficiently reversed with neostigmine and glycopyrrolate. Despite repeated doses, this patient needed to be reintubated and sedated until spontaneous recovery was observed over the next few hours. With the increasing preference to utilize sugammadex in the perioperative setting, we are starting to notice a significant number of patients displaying residual paralysis despite sugammadex administration as a solo or rescue agent at our institution. We believe that more attention is needed to examine the factors that contribute to insufficient neuromuscular blockade reversal in these settings.

## Introduction

Postoperative residual neuromuscular block (NMB) from rocuronium following administration of reversal agents is an anesthetic complication that needs monitoring to ensure patient safety [1]. In the situation of recurarization after reversal with neostigmine and glycopyrrolate, sugammadex has been shown to be an effective rescue agent [2-5]. However, usage in this scenario has not been properly evaluated and may have unintended side effects beyond what is known when these agents are administered independently [6-10]. Furthermore, more research is needed to determine elements that contribute to sugammadex failure in this setting, and how they compare to factors known to negatively affect independent sugammadex usage: insufficient reversal, concurrent medical therapies, and patient-related factors [6,11-16]. Sugammadex usage in general also requires more safety data in certain populations, such as pregnant patients or those with severe renal impairment [6]. We report a case of an 81-year-old man with unresolved weakness after receiving both reversal agents, reinforcing the importance of continued patient monitoring in the perioperative period and the need to research sugammadex emergency rescue further.

## Case presentation

An 81-year-old man (75 kg, 175 cm, body mass index (BMI) 24.5) with a history of persistent atrial fibrillation, coronary artery disease, stable thoracic aortic aneurysm, and sensorimotor polyneuropathy following prior spinal surgery underwent holmium laser enucleation of the prostate under general anesthesia. His medications included tamsulosin, losartan, isosorbide mononitrate, hydralazine, furosemide, carvedilol and apixaban. His past surgical history included lumbar laminectomy and open reduction internal fixation of the femur with no notable anesthetic complications. Preoperative labs showed normal electrolytes, potassium at 4.4 mmol/L, blood urea nitrogen at 12 mg/dL, creatinine at 0.8 mg/dL, and estimated glomerular filtration rate greater than 60 mL/min/1.73 m². During both procedures, muscle relaxation was achieved with rocuronium and was reversed with sugammadex. His physical examination revealed anasarca. 

Anesthesia was induced with propofol 150 mg, rocuronium 50 mg, fentanyl 50 mcg, and lidocaine 50 mg. Rocuronium is the most readily available neuromuscular blocking agent at our hospital and was chosen due to the lack of both contraindications or complication history in this patient. Muscle relaxation was maintained throughout the case with an additional rocuronium 50 mg (total 1.3 mg/kg), and extent of neuromuscular blockade was quantitatively monitored using an EMG monitor (TetraGraph®, Senzime AB, Uppsala, Sweden). Sevoflurane was administered with a minimum alveolar concentration goal of 1.2. At the end of the 180-minute procedure, neostigmine 5 mg (0.07 mg/kg) IV and glycopyrrolate 0.5 mg IV were administered and the patient was extubated after demonstrating spontaneous eye opening and ability to obey commands. Sevoflurane levels were undetectable, and no magnesium was given.

Despite being awake and alert, the patient began to display rapid, shallow breathing and desaturation within 15 minutes of extubation. Attempted mask ventilation proved to be difficult, leading to a presumptive diagnosis of laryngospasm. Propofol 50 mg and succinylcholine 20 mg were given to treat the presumed laryngospasm. Mask ventilation improved; however, the patient continued to have poor ventilatory effort. Train of four (TOF) monitoring on the ulnar nerve revealed three twitches with fade. At this point, laryngeal mask airway was placed to assist with ventilation. Suspecting incomplete reversal of NMB, decision was made to administer sugammadex 150 mg, as maximal amount of neostigmine was already given and further dosage would introduce the risk of precipitating a cholinergic crisis. With no evidence of improvement in muscle strength and TOF testing, we chose to administer an additional 150 mg of sugammadex. The muscle weakness persisted as evidenced with minimal improvement on TOF. Another 250 mg of sugammadex was given (total 7.3 mg/kg). Due to the unknown nature of the patient’s persistent neuromuscular weakness, the patient was intubated, sedated on a propofol infusion, and transported to the post-anesthesia care unit (PACU). Immediate and follow-up arterial blood gas showed no abnormalities. Over the next several hours, the patient spontaneously regained his muscle strength and was successfully extubated in the PACU. The patient fully recovered without further complication and was discharged the next day.

## Discussion

Postoperative residual NMB from rocuronium and its associated complications are well documented in the literature [1]. Even with administration of reversal agents, many patients continue to display TOF ratios <0.9 upon arrival in the PACU. This phenomenon appears to occur more frequently in older patients because of geriatric physiological changes such as decreased renal function and changes in acetylcholine or acetylcholine receptor concentrations. Although newer agents have become normalized into clinical practice, neostigmine continues to be used as a widely available pharmacological reversal of NMB. Additional rescue administration of sugammadex, as noted in this case, has been shown to eliminate any residual action of rocuronium [2-5].

The recommended dosing of sugammadex after rocuronium administration is 2 mg/kg of actual body weight (ABW) when at least two responses are obtained to TOF stimulation, 4 mg/kg when spontaneous twitch response has reached one to two post-tetanic counts (PTC) without twitch response to TOF stimulation, and up to 16 mg/kg when rapid reversal of profound NMB is required [6]. Dosing according to ABW instead of ideal body weight has shown to have significant faster recovery in patients with morbid obesity (BMI>40), suggesting a potential need for greater than recommended dosing of sugammadex to account for increased rocuronium requirements. Due to variability between patients, quantitative twitch monitoring is important to ensure proper recovery in case a greater than recommended dose is required. This variability can also be observed within the same patient following physiologic changes such as renal function. The patient in this case demonstrated no decline in preoperative renal function when compared to previous surgeries. Sugammadex has shown to produce rapid and dose-dependent recovery from high-dose rocuronium-induced (up to 1.2 mg/kg) NMB. While the total rocuronium dosage (1.3 mg/kg) in our case exceeds what has been tested in the past, previous studies initiated reversal after a much shorter duration of NMB, allowing for less metabolism and clearance of rocuronium. Furthermore, the sugammadex in our case was administered as a rescue agent following failed reversal with neostigmine/glycopyrrolate rather than instead of a sole, initial reversal agent.

Sugammadex is neither indicated nor approved for emergency rescue of patients who fail adequate NMB reversal with neostigmine [6]. However, it is frequently used as a first-line agent to deal with this critical situation. More data is needed on its safety, as patients who receive a rescue administration of sugammadex following reversal with neostigmine/glycopyrrolate have been shown to be at greater risk for postoperative pulmonary complications [7]. One major concern about this method is that neostigmine administered in the absence of NMB can lead to clinical signs of re-paralysis, and that unopposed acetylcholine after rocuronium removal with sugammadex could result in a similar effect [3]. There are other cases in the literature describing this application in practice, in which failed NMB reversal with neostigmine was successfully rescued with sugammadex 2-4 mg/kg, but no cases could be found like in this case, where further intervention using up to 7.3 mg/kg was unsuccessful [2-5].

Since its introduction as an alternative agent to reverse NMB, sugammadex has been studied extensively and compared with acetylcholine inhibitors such as neostigmine [1]. Sugammadex selectively binds with high affinity to free rocuronium molecules in a one-to-one ratio, forming an inactive complex that is eliminated from the body renally [6]. In the setting of renal insufficiency, previous studies have shown no significant difference in the ability of sugammadex to reverse NMB without dosing adjustment, but decreased clearance can lead to considerably more sugammadex exposure [6].

As sugammadex does not influence plasma cholinesterase levels or muscarinic receptors, there are fewer serious adverse effects linked to potential overdose than with anticholinergics [8]. This includes postoperative urinary retention, hemodynamic changes, double vision, and dry mouth [9]. Furthermore, patients with respiratory pathologies have shown decreased incidence of post-operative respiratory complications when treated with sugammadex over neostigmine [10]. Previous research has shown that NMB reversal with sugammadex is significantly faster than reversal with neostigmine, but this decreased time to extubation has questionable clinical significance on time to discharge from the PACU [17]. While this decreased time in the operating room has shown to be insignificant for increased scheduling of surgical procedures, perhaps this time could be more meaningful in reducing operating room costs or in trauma centers when staffing is limited. Due to the safety that sugammadex provides over other alternatives, anesthesiologists should consider using sugammadex as a first-line therapy in patients with greater risk for anesthetic complications.

It is important to note that recurarization can still occur when initial reversal of NMB is performed using sugammadex [11]. This can be affected by factors such as insufficient dosing, concurrent medical therapies, or patient-related factors. There are few endogenous and exogenous compounds that have shown potential affinity with sugammadex and resulting delay of NMB reversal: flucloxacillin, fusidic acid, toremifene, and steroid compounds [6,12]. Of these, only toremifene displayed clinical significance in recovery time. Other medical therapies that can contribute to postoperative muscle weakness include induced hypothermia, magnesium sulfate, and residual sevoflurane [13,14]. Patient-related factors with a similar effect are hypokalemia and myasthenia gravis [15,16]. It is possible for patients with no known history of myasthenia gravis to be in the process of developing the disease at the time of surgery and receive the diagnosis afterwards [16]. In this situation, a neurology consult could be placed to exclude myasthenia gravis for future anesthesia events. For patients with myasthenia gravis, sugammadex is preferred over acetylcholinesterase agents for NMB reversal and has shown to work as a rescue agent after neostigmine administration [16]. However, more research needs to be done about its efficacy in this scenario.

In certain cases, impaired consciousness or spontaneous ventilation can occur despite normal accelomyographic monitoring, so physicians should retain some reservation when using TOF as a measure of detecting residual blockade, especially in patients with higher risk [13]. Just like in resolving postoperative residual curarization with initial neostigmine use, further pharmacologic intervention with sugammadex has shown to improve symptoms. As overdosage of sugammadex is not particularly problematic, the risk of underdosage should not warrant a delay in intervention when there is suspicion of residual blockade [18].

## Conclusions

This case report demonstrates the necessity for neuromuscular function monitoring after using muscle relaxants for general anesthesia. Continued monitoring to avoid excessive NMB and ensure success of pharmacological reversal is recommended, and early intervention with the administration of sugammadex may reduce the severity and worsening of anesthetic events. However, it is important to keep in mind that despite the great efficacy of sugammadex, there are many factors that can lead to its failure. For instance, we cannot rule out completely the possibility that this patient's resistant postoperative weakness was an early sign of newly developing pathology, such as myasthenia gravis. More research is needed to examine such factors in order to improve patient safety in the post-operative period. We hypothesize that one of these is a variability in patient receptor affinity to rocuronium, in which these receptors compete more strongly with sugammadex and prolong the neuromuscular blockade.
